# Systematic Theoretical Study on the pH-Dependent Absorption and Fluorescence Spectra of Flavins

**DOI:** 10.3390/molecules28083315

**Published:** 2023-04-08

**Authors:** Jinyu Wang, Yajun Liu

**Affiliations:** 1 Key Laboratory of Theoretical and Computational Photochemistry, Ministry of Education, College of Chemistry, Beijing Normal University, Beijing 100875, China; 2 Center for Advanced Materials Research, Beijing Normal University, Zhuhai 519087, China

**Keywords:** flavin, pH, spectra, DFT

## Abstract

Flavins are a class of organic compounds with the basic structure of 7,8-dimethy-10-alkyl isoalloxazine. They are ubiquitous in nature and participate in many biochemical reactions. Due to various existing forms, there is a lack of systematic research on the absorption and fluorescence spectra of flavins. In this study, employing the density functional theory (DFT) and time-dependent (TD) DFT, we calculated the pH-dependent absorption and fluorescence spectra of flavin of three redox states (quinone, semiquinone, and hydroquinone) in solvents. The chemical equilibrium of three redox states of flavins and the pH effect on the absorption spectra and fluorescence spectra of flavins were carefully discussed. The conclusion helps with identifying the existing forms of flavins in solvent with different pH values.

## 1. Introduction

**Figure 1:**
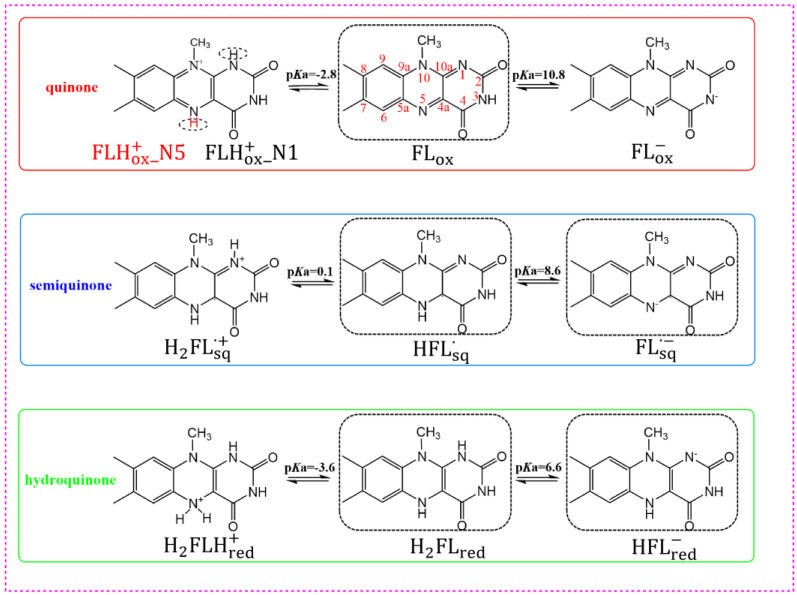
Structures and acid-base equilibrium of flavins and labels. The molecules in the black dotted box are the forms that exist under physiological conditions.

Flavins are highly active molecules used by a variety of enzymes to perform a number of biological functions, including biocatalysis [1,2,3], biosynthesis [4], bioluminescence [5,6,7], and so on. Among flavins, flavin mononucleotide (FMN) and flavin adenine dinucleotide (FAD) are two common flavin molecules with enzymatic activity [8]. Their basic structures are 7, 8-dimethyl isoalloxazine, which has the potential to transfer single and double electrons. Therefore, flavins exist in three different redox states: an oxidized state (quinone), one-electron-reduced state (semiquinone), and two-electron-reduced state (hydroquinone). For quinone, semiquinone, or hydroquinone, acid-base equilibrium involves a cationic form, neutral form, and anionic form. Notably, the cationic quinone form in the ground (S_0_) and the first singlet excited (S_1_) states have different protonated species, which are cationic oxidized form ${FLH}_{ox}^{+}\_N1$ and ${FLH}_{ox}^{+}\_N5$, respectively [9]. The structures and acid-base equilibrium of flavins are shown in Figure 1.

Flavins have been widely studied in various forms due to their versatility [10,11,12,13]. Different forms of flavins have characteristic UV-visible absorption spectra, which can be inversely used to identify flavin. The UV-visible absorption spectra of flavins have been studied in gas [14], solutions [14,15,16,17], and protein [12,18]. However, due to the instability of flavin semiquinone, generally the absorption spectra of only flavin quinone or flavin hydroquinone have been studied in experimental studies [15,16]. The absorption spectra of five flavin forms existing under the physiological conditions shown in Figure 1 were studied theoretically [17]. The fluorescence spectra of flavins have also been studied as another tool to characterize their photophysical properties [19,20,21,22]. Flavin quinone is highly fluorescent, and the fluorescence of FAD can be used for autofluorescence imaging to monitor for subcellular activity [22]. The anionic semiquinone ${FMN}^{∙-}$ in the nitronate monooxygenase exhibits no fluorescence, whereas the neutral semiquinone radical ${FMNH}^{∙}$ shows a relatively strong fluorescence [23]. Flavin hydroquinone shows weak fluorescence in solution, but is enhanced in a rigid protein environment [12]. To sum up, previous studies on the absorption spectra and fluorescence spectra of flavin have involved only limited forms of flavin. It is necessary to study the absorption and fluorescence spectra of other forms of flavin. Therefore, there is still a lack of systematic research on the absorption spectra and fluorescence spectra of various forms of flavins. In this study, we systematically studied the absorption and fluorescence spectra of the ten forms of flavins shown in Figure 1 in aqueous solution. In addition, the chromophore of the reduced flavin was found to be very sensitive to variations in the pH of the solvent [12]. To consider the pH effect on spectra of flavins, the pKa of the S_0_-state and ${pKa}^{*}$ of the S_1_-state flavin were calculated, which was helpful to confirm whether the flavin was a photoacid. For photoacids, the ${pKa}^{*}$ was considerably less than the p*K*a. Moreover, based on the pKa and ${pKa}^{*}$ of each flavin, the chemical equilibrium of the three-redox state of the flavin was studied, and pH-dependent absorption spectra and fluorescence spectra were simulated.

## 2. Computational Details

***Quantum mechanics (QM) calculations.*** The density functional theory (DFT) [24,25] and the time-dependent (TD) DFT [26,27] were employed. Functionals, basis sets, and solvent models were tested based on the reaction ($H_{2}{FL}_{red}$ → ${HFL}_{red}^{-}$ + $H^{+}$) with an experimental pKa of 6.5 [28,29]. As the test results in Table S1 show, the M06-2X [30] /6-31 + G** level with solvation model density (SMD) approach [31] provided sufficient precision for calculating pKa. Therefore, all calculations in the S_0_ state were carried out at the M06-2X/6-31 + G** computational level in this paper. In addition, the SMD approach considering the polarity and nonpolarity was adapted to simulate the solution conditions. The geometries of the ten forms of flavin in Figure 1 were optimized at the M06-2X/6-31 + G** level for the S_0_ state in gas and water. Gibbs free energies were obtained from the vibrational analysis. For the photophysical properties of flavin, the B3LYP is usually employed for calculating the spectra and behaves well [17,18]. For radicals and anions, a relatively big basis set should be used. Therefore, the vertical absorption spectra of nine forms of flavin in water in the S_0_ state were predicted at the TD B3LYP/6-311 ++G** level, and 20 excited states were involved. Solvent relaxation was not included during excitation. The geometries of nine forms of flavin in the S_1_ state in water were optimized, and their fluorescence spectra were predicted at the TD B3LYP/6-311 ++G** level. All the above calculations were performed by the Gaussian09 program package [32].

**Figure 2:**
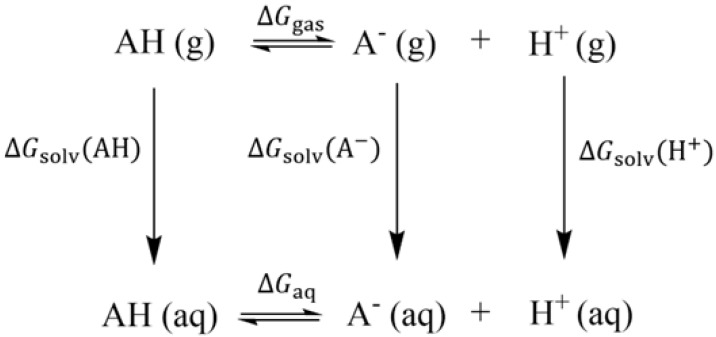
Born–Haber thermodynamic cycle.

***Calculation of the*** pKa ***and*** ${pKa}^{*}$. The ability of AH to deprotonate to A^−^ can be expressed by the negative logarithm of the acid constant of AH, which is pKa for the S_0_ state and ${pKa}^{*}$ for the S_1_ state. pKa is related to the dissolution free energy $∆G_{aq}$, which can be computed by the Born–Haber thermodynamic cycle [33] (see Figure 2). The specific steps are as follows [34]:

(1) $$∆G_{gas}=G_{gas}^{AH}+G_{gas}^{A^{-}}+G_{gas}^{H^{+}}$$

where $∆G_{gas}$ refers to the variation of the Gibbs free energy of the gas phase. $G_{gas}^{AH}$ and $G_{gas}^{A^{-}}$ refer to the Gibbs free energy of AH and A^−^, respectively, which can be obtained by vibration analysis of the optimized optimal geometric configuration of AH and A^−^. $G_{gas}^{H^{+}}$ refers to the free energy of the proton in gas phase (−6.28 kcal/mol) [35].

(2) $$∆G_{aq}=∆G_{gas}-∆G_{solv}\left(AH\right)+∆G_{solv}\left(A^{-}\right)+∆G_{solv}\left(H^{+}\right)+∆G_{add}$$

where $∆G_{solv}\left(AH\right)$ refers to the solvation free energy of AH, calculated from the variation of the single-point energy between the gas and water. Similarly, $∆G_{solv}\left(A^{-}\right)$ refers to the solvation free energy of A^−^, calculated from the variation of the single-point energy between the gas and water. $∆G_{solv}\left(H^{+}\right)$ is the solvation free energy of the hydrogen ion (−265.9 kcal/mol) [36], and $∆G_{add}$ represents the transfer of a solute molecule from the 1 atm gas phase to the 1 M solvent standard state (1.89 kcal/mol).

(3) $$pKa=\frac{∆G_{aq}}{2.303RT}$$

where *R* refers to the molar constant of gas, which is valued at 8.314 J/(mol/K). *T* is the temperature, which is valued at 298.15 K.

${pKa}^{*}$ can be obtained by the following equation [37]:

(4) $$pKa^{*}=pKa+\frac{\Delta E_{abs}}{2.303RT}$$

where $\Delta E_{abs}$ is the absorption energy variation between protonated (AH) and anionic ($A^{-}$) species.

***Calculation of the relative concentration and the pH-dependent spectra.*** The relative concentration of each form of flavins in the S_0_ state at a specific pH can be calculated with the pKa. The calculation process is as follows:

(5) $${AH}_{2}^{+}\to AH+H^{+}\;K_{a_{1}}=\frac{\left[AH\right]\left[H^{+}\right]}{AH_{2}^{+}}$$

(6) $$AH\to A^{-}+H^{+}\;K_{a_{2}}=\frac{\left[A^{-}\right]\left[H^{+}\right]}{\left[AH\right]}$$

where ${AH}_{2}^{+}$, AH, and $A^{-}$ represent the cation, neutral molecule, and anion in the same redox state, respectively. $K_{a_{1}}$ and $K_{a_{2}}$ are the acid constant of ${AH}_{2}^{+}$ and AH, respectively.

Then the concentration ratio of three forms of each redox state can be presented as follows:

(7) $$[{AH}_{2}^{+}]:[AH]:[A^{-}]=1:[H^{+}{]}^{-1}K_{a_{1}}:[H^{+}{]}^{-2}{K_{a_{1}}K}_{a_{2}}$$

Then the relative concentrations of the three chemical forms for each redox state at a specific pH value can be obtained, and the pH-dependent absorption spectra can be simulated. At a specific pH value, multiply the relative concentrations of the three forms by the wavelengths of the three forms of absorption spectrum to obtain the wavelength at that pH value, and multiply the relative concentrations of the three forms by the intensity of the three forms of absorption spectrum to obtain the intensity at the pH value. The same method is used for obtaining absorption spectra at other pH values. These spectra at different pH values can be integrated to obtain pH-dependent absorption spectra.

Similarly, the relative concentration of each form of flavins in the S_1_ state at a specific pH can be calculated with the ${pKa}^{*}$. In addition, the pH-dependent fluorescence spectra can be simulated by the same method with the pH-dependent absorption spectra.

## 3. Results and Discussion

### 3.1. Absorption and Fluorescence Spectra of Flavins in Solution

**Figure 3:**
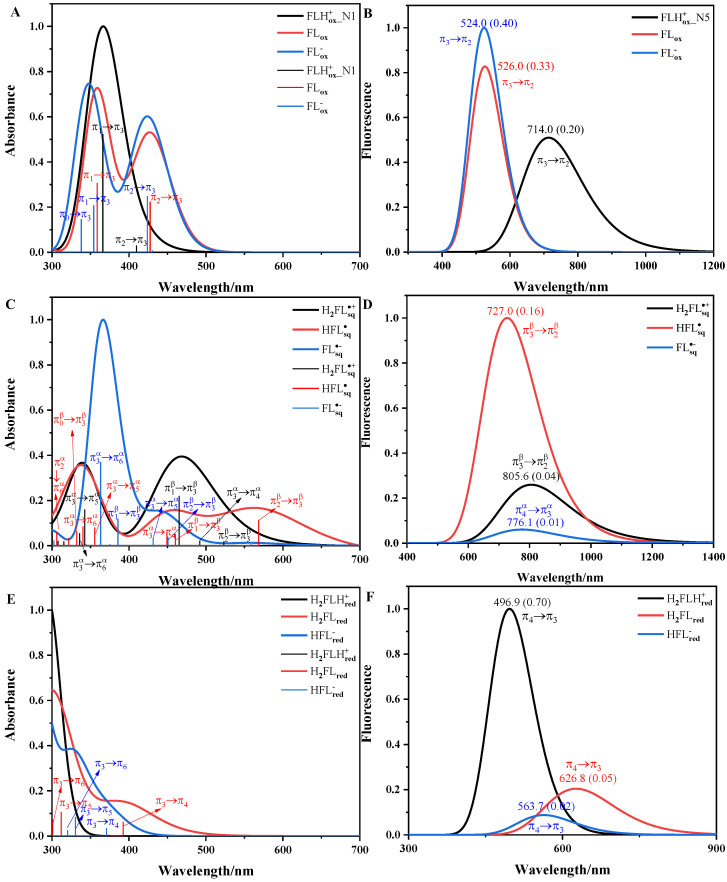
The computational absorption spectra (**A**,**C**,**E**) and fluorescence spectra (**B**,**D**,**F**) of flavin quinone, flavin semiquinone, and flavin hydroquinone in aqueous solution. Thin vertical lines represent the electronic excited states, with the corresponding transition analysis next to them. Values in parentheses represent the *f* of fluorescence spectra.

The calculated vertical absorption spectra and fluorescence spectra of the ten forms of flavins in solution are shown in Figure 3. The TD state order, transition, absorption maximum ($\lambda_{max}$), and oscillator strength (*f*) of the absorption spectra of the flavins are listed in Table S2. The full width at half-maximum (FWHM) of the spectra were determined empirically and are listed in Table S3. The spectra were normalized, no scaling factor was used, and the electronic excited states and their corresponding transition analysis next to them are shown in Figure 3. The frontier molecular orbital $\pi_{3}$ plays a central role for all redox forms of flavin; it mainly delocalizes over the isoalloxazine ring and exhibits a bonding character between C4a and C10a and an anti-bonding character between N10 and C10a. The π orbitals with energy higher than $\pi_{3}$ are $\pi_{4},\pi_{5},\pi_{6}\ldots$, and those with energy lower than $\pi_{3}$ are $\pi_{2},\pi_{1},\pi_{0}\ldots$. These definitions and sequences are consistent with previous studies [18,38].

For flavin quinone, $\pi_{3}$ is the lowest unoccupied molecular orbital (LUMO). In Figure 3A, the absorption spectrum of ${FLH}_{ox}^{+}\_N1$ has an absorption peak, which comes from the contribution of the $\pi_{2}\to \pi_{3}$ transition from the S_0_ state to the S_1_ state and the $\pi_{1}\to \pi_{3}$ transition from the S_0_ state to the S_2_ state. The $\pi_{1}\to \pi_{3}$ transition corresponds to the $\lambda_{max}$ at 366.0 nm, corresponding to the experimental maximum ($\lambda_{exp}$) around 394.0 nm [15]. The absorption spectrum of ${FL}_{ox}$ has two absorption peaks: the lowest-energy peak is represented by the $\pi_{2}\to \pi_{3}$ transition from S_0_ to S_1_, and the highest-energy peak is represented by the $\pi_{1}\to \pi_{3}$ transition from S_0_ to S_2_. The $\pi_{2}\to \pi_{3}$ and the $\pi_{1}\to \pi_{3}$ transitions correspond to the $\lambda_{max}$ values at 427.5 and 358.7 nm, corresponding to the $\lambda_{exp}$ values around 445.0 and 370.0 nm [15,39], respectively. The absorption spectrum of ${FL}_{ox}^{-}$ also has two peaks: the lowest-energy peak is mainly represented by the $\pi_{2}\to \pi_{3}$ transition from S_0_ to S_2_, and the highest-energy peak involves the $\pi_{1}\to \pi_{3}$ transition from S_0_ to S_4_ and the $\pi_{0}\to \pi_{3}$ transition from S_0_ to S_5_. The $\pi_{1}\to \pi_{3}$ transition and $\pi_{0}\to \pi_{3}$ transition correspond to the $\lambda_{max}$ values at 424.0 and 354.2 nm, corresponding to the $\lambda_{exp}$ values of 450.0 and 350.0 nm [15], respectively. The absorption spectra of ${FLH}_{ox}^{+}\_N1$, ${FL}_{ox}$ and ${FL}_{ox}^{-}$ all reproduce the spectral characteristics (single-peaked spectrum for ${FLH}_{ox}^{+}\_N1$, double-peaked spectra for ${FL}_{ox}$ and ${FL}_{ox}^{-}$). In Figure 3B, the transitions from S_1_ to S_0_ for ${FLH}_{ox}^{+}\_N5$, ${FL}_{ox}$, and ${FL}_{ox}^{-}$ are $\pi_{3}\to \pi_{2}$ transitions, and the $\pi_{3}\to \pi_{2}$ transition for ${FL}_{ox}$ corresponds to the fluorescence wavelength ($\lambda_{F})$ at 526.0 nm, closely reproducing the experimental value around 530.0 nm [15]. The large *f* of flavin quinone is consistent with the experimental high bright fluorescence of oxidized flavin [39].

For flavin semiquinone, $\pi_{3}$ is a singly occupied molecular orbital (SOMO). In Figure 3C, the lowest-energy peak of the absorption spectrum of ${H_{2}FL}_{sq}^{∙+}$ mainly involves the contributions of the $\pi_{2}^{\beta }\to \pi_{3}^{\beta }$, $\pi_{3}^{\alpha }\to \pi_{4}^{\alpha }$, and $\pi_{1}^{\beta }\to \pi_{3}^{\beta }$ transitions from the S_0_ state to the first three excited singlet states. The highest-energy peak mainly comes from the contributions of the $\pi_{3}^{\alpha }\to \pi_{5}^{\alpha }$ and $\pi_{3}^{\alpha }\to \pi_{6}^{\alpha }$ transitions from the S_0_ state to the S_5_ state and the S_6_ state. The triple-peaked absorption spectra of ${HFL}_{sq}^{∙}$ involves many electronic transitions, where the bright $\pi_{2}^{\beta }\to \pi_{3}^{\beta }$, $\pi_{1}^{\beta }\to \pi_{3}^{\beta }$, and $\pi_{3}^{\alpha }\to \pi_{6}^{\alpha }$ transitions are found at 568.6, 460.3, and 340.2 nm, respectively, corresponding to the $\lambda_{exp}$ around 571.0, 481.0, and 340.0 nm, respectively [40]. For ${FL}_{sq}^{∙-}$, the $\pi_{2}^{\beta }\to \pi_{3}^{\beta }$ transition and the $\pi_{3}^{\alpha }\to \pi_{6}^{\alpha }$ transition correspond to the $\lambda_{max}$ values at 450.8 and 363.1 nm, corresponding to the $\lambda_{exp}$ around 480.0 and 370.0 nm, respectively [40]. There is no experimental report on the absorption spectra of ${H_{2}FL}_{sq}^{∙+}$. The absorption spectra of ${HFL}_{sq}^{∙}$ and of ${FL}_{sq}^{∙-}$ well reproduce the experimental spectral characteristics (triple-peaked spectrum for ${HFL}_{sq}^{∙}$ and double-peaked spectrum for ${FL}_{sq}^{∙-}$). In Figure 3D, the transitions from S_1_ to S_0_ are the $\pi_{3}^{\beta }\to \pi_{2}^{\beta }$ transitions for ${H_{2}FL}_{sq}^{∙+}$ and ${HFL}_{sq}^{∙}$ and the $\pi_{4}^{\alpha }\to \pi_{3}^{\alpha }$ transition for ${FL}_{sq}^{∙-}$. The *f* of ${HFL}_{sq}^{∙}$ is large, and it is much larger than that of ${FL}_{sq}^{∙-}$. This is consistent with the results that flavin semiquinone yielded in the nitronate monooxygenase [23]. Due to flavin semiquinone not being stable in solution and being a transient species in the excited state, there is no experimental study on the fluorescence spectra of flavin semiquinone in aqueous.

For flavin hydroquinone, $\pi_{3}$ is the highest occupied molecular orbital (HOMO). For $H_{2}{FLH}_{red}^{+}$, the $\lambda_{max}$ corresponding to the $\pi_{3}\to \pi_{4}$ transition from S_0_ to S_1_ is 295.6 nm. Therefore, there is no absorption peak for $H_{2}{FLH}_{red}^{+}$ in Figure 3E. In Figure 3E, the absorption spectra of both $H_{2}{FL}_{red}$ and ${HFL}_{red}^{-}$ have a peak [39]. For $H_{2}{FL}_{red}$, the absorption peak comes from the contribution of the $\pi_{3}\to \pi_{4}$ transition, which corresponds to the $\lambda_{max}$ at 392.4 nm, matching the $\lambda_{exp}$ around 395.0 nm. For ${HFL}_{red}^{-}$, the $\pi_{3}\to \pi_{5}$ transition corresponds to the $\lambda_{max}$ at 330.4 nm, corresponding to the $\lambda_{exp}$ around 342.0 nm [39]. In Figure 3F, the transitions from S_1_ to S_0_ for $H_{2}{FLH}_{red}^{+}$, $H_{2}{FL}_{red}$, and ${HFL}_{red}^{-}$ are $\pi_{4}\to \pi_{3}$ transitions, which correspond to the $\lambda_{F}$ values at 496.9, 626.8, and 563.7 nm, respectively. $H_{2}{FLH}_{red}^{+}$ does not exist in the pH range of 1~14. In addition, the *f* of $H_{2}{FL}_{red}$ and ${HFL}_{red}^{-}$ is low. All these are consistent with the experimental result of flavin hydroquinone that $\lambda_{F}$ is not below 500 nm and essentially nonfluorescent in aqueous solutions [39].

In summary, our calculation results can almost reproduce the existing experimental values. As listed in Rajiv K. Kar’s review [41], some theoretical studies have calculated the vertical excitation energy (${∆E}_{ex}$) of flavins [7,8,42,43,44]. The ${∆E}_{ex}$ and *f* of the five flavin forms (${FL}_{ox}$, ${HFL}_{sq}^{∙}$, ${FL}_{sq}^{∙-}$, $H_{2}{FL}_{red}$, and ${HFL}_{red}^{-}$) in aqueous solution are calculated at the level of TD B3LYP/def2-TZVPP [38] and TD B3LYP/cc-pVTZ [17]. The comparison of our calculation results with their reported results and the experimental values are listed in Table 1. For all five forms of flavin, the $\lambda_{max}$ values we predicted were closer to the $\lambda_{exp}$ than those predicted in Ref. [17] and Ref [38]. For ${FL}_{ox}$, $H_{2}{FL}_{red}$, and ${HFL}_{red}^{-}$, the transitions corresponding to the $\lambda_{exp}$ were completely consistent with Ref. [38]. For ${HFL}_{sq}^{∙}$ and ${FL}_{sq}^{∙-}$, the transitions corresponding to the $\lambda_{exp}$ of 340.0 and 370.0 nm involved in the highest peak of spectra were $\pi_{3}^{\alpha }\to \pi_{6}^{\alpha }$ transitions, which were different from the $\pi_{0}^{\beta }\to \pi_{3}^{\beta }$ transition and the $\pi_{1}^{\beta }\to \pi_{3}^{\beta }$ transition reported in Ref. [38]. The $\pi_{0}^{\beta }\to \pi_{3}^{\beta }$ transition for ${HFL}_{sq}^{∙}$ and the $\pi_{1}^{\beta }\to \pi_{3}^{\beta }$ transition for ${FL}_{sq}^{∙-}$ in Table S2 were found to have lower *f* than those of the $\pi_{3}^{\alpha }\to \pi_{6}^{\alpha }$ transition. The $\lambda_{max}$ and *f* predicted in this paper, in most cases, were similar to those predicted in Ref. [17], but the corresponding excited state orders were different. All these differences may be due to the computational level and solvation model. In addition, the lowest-energy peak of the absorption spectra of ${FL}_{ox}$, ${HFL}_{sq}^{∙}$ ${FL}_{sq}^{∙-},and{HFL}_{red}^{-}$ involved the contributions of the same electronic transition as those in Ref. [18].

### 3.2. Chemical Equilibrium of Flavins in Solution

**Figure 4:**
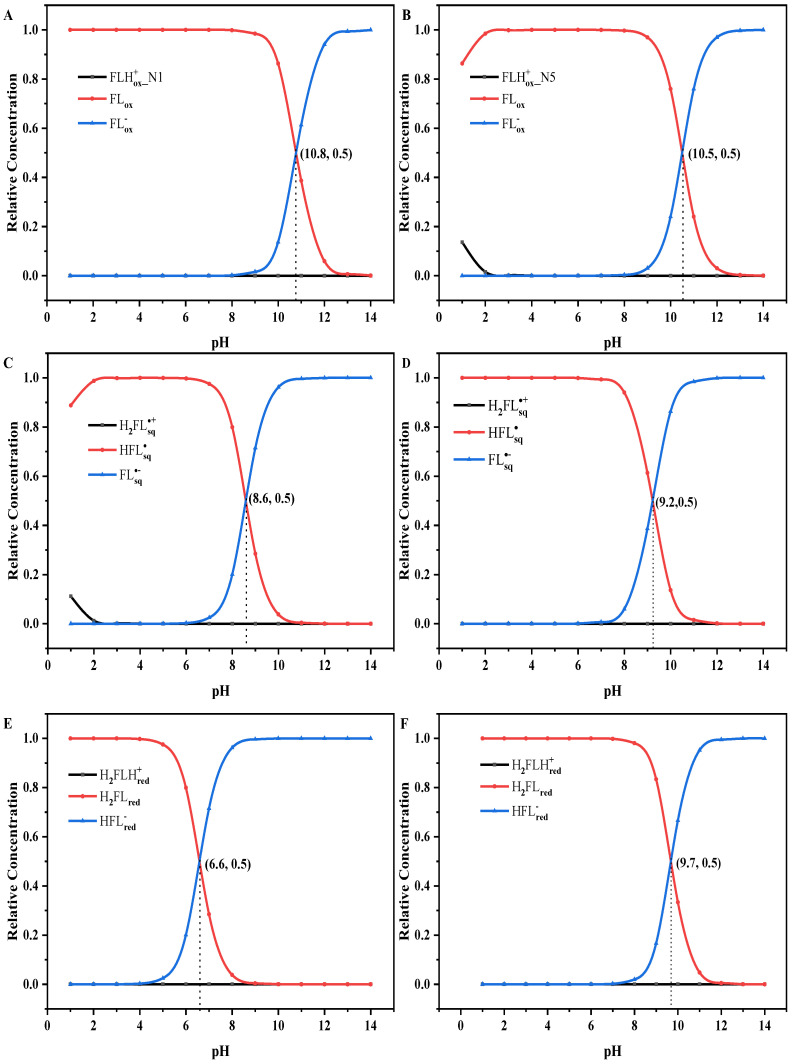
The diagrams of relative concentrations for the S_0_ state (**A**,**C**,**E**) and S_1_ state (**B**,**D**,**F**) of different forms of flavins in aqueous solution at different pH values.

The proton affinity of flavins is easily affected by the pH of the solution. To confirm the existence forms of flavin at different pH values, the calculated (calc.) and experimental (exp.) pKa and $pKa^{*}$ values are listed in Table 1. All calculated pKa and $pKa^{*}$ values of flavins are in good agreement with the reported experimental values. In addition, the $pKa^{*}$ value of ${FLH}_{ox}^{+}\_N5$ in the S_1_ state is greater than the pKa value of ${FLH}_{ox}^{+}\_N1$ in the S_0_ state. This shows that the acidity of the oxidized cationic form in the S_1_ state is weaker than that in the S_0_ state, which is consistent with the previous research results [9]. This can indicate that the cationic quinone is not a photoacid but a photobase. From Table 2, we can see that the $pKa^{*}$ values of ${H_{2}FL}_{sq}^{∙+}$ and $H_{2}{FLH}_{red}^{+}$ are less than their pKa values, which indicates that they are photoacids. By contrast, the $pKa^{*}$ values of ${HFL}_{sq}^{∙}$, $H_{2}{FL}_{red}$, and cationic quinone are greater than their pKa values, which indicates that they are photobases. There is little difference between the $pKa^{*}$ value and pKa value of ${FL}_{ox}.$ Based on the pKa and $pKa^{*}$, the relative concentrations of each form of flavins in the S_0_ state and S_1_ state at different values are listed in Tables S4 and S5, respectively. In addition, the diagrams of the relative concentrations of different forms of flavins in the S_0_ state and S_1_ state with various pH values are shown in Figure 4. For the flavin quinone (Figure 4A,B), in the S_0_ state, ${FLH}_{ox}^{+}\_N1$ almost does not exist in the entire pH range. When the pH value is less than 8, ${FL}_{ox}$ is the dominant form. When the pH is larger than 8, the relative concentration of ${FL}_{ox}$ decreases gradually with the increase of pH, and the relative concentration of ${FL}_{ox}^{-}$ increases gradually, and they are equal at the pH value of 10.8. Later, with the pH increasing, ${FL}_{ox}^{-}$ gradually becomes the dominant form. Different from the S_0_ state, ${FLH}_{ox}^{+}\_N5$ in the S_1_ state exists in the pH range of 1.0~2.1. For the flavin semiquinone (Figure 4C,D), ${H_{2}FL}_{sq}^{∙+}$ exists when the pH is less than 2 for the S_0_ state but does not exist in the whole range of pH for the S_1_ state. For the S_0_ and S_1_ states, ${HFL}_{sq}^{∙}$ are the dominant forms when the pH is less than 6 and 7, and when the pH is larger than 6 and 7, the relative concentration of ${HFL}_{sq}^{∙}$ gradually decreases, and the relative concentration of ${FL}_{sq}^{∙-}$ gradually increases. They reach equilibrium when the pH is 8.6 and 9.2 for the S_0_ state and the S_1_ state, respectively. Then, with the increase of the pH, ${FL}_{sq}^{∙-}$ gradually becomes the dominant form for both the S_0_ and S_1_ states. For the flavin hydroquinone (Figure 4E,F), the cationic form $H_{2}{FLH}_{red}^{+}$ does not exist in the whole pH range for both the S_0_ and S_1_ states. The neutral form $H_{2}{FL}_{red}$ and the anionic form ${HFL}_{red}^{-}$ are the dominant forms before and after they reach equilibrium, respectively.

### 3.3. The pH-Dependent Absorption and Fluorescence Spectra of Flavins in Solution

**Figure 5:**
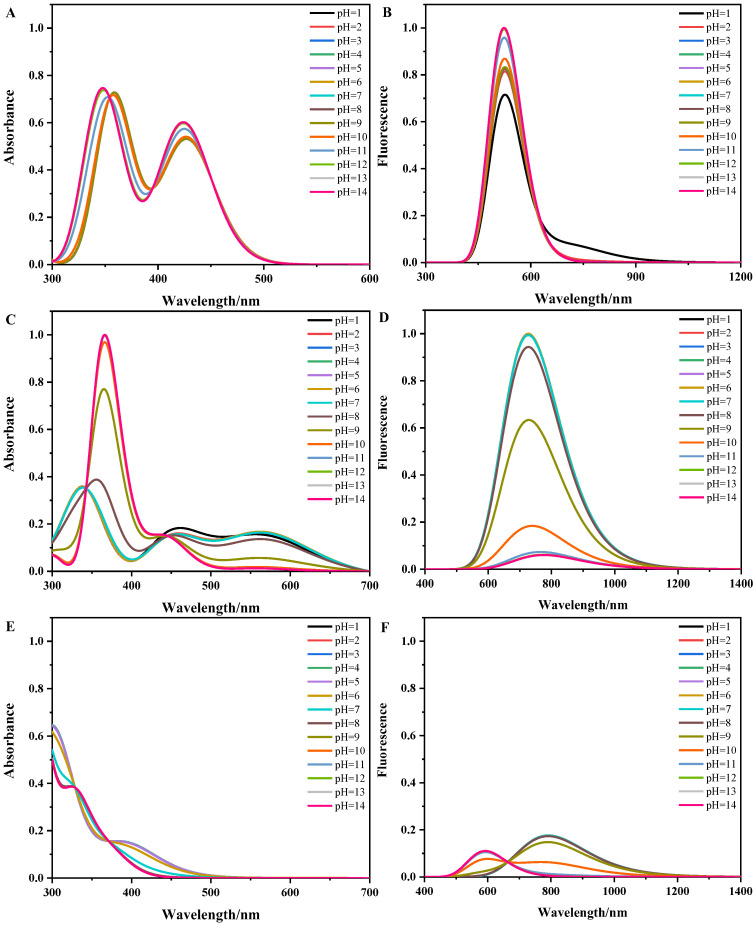
The pH-dependent absorption (**A**,**C**,**E**) and fluorescence (**B**,**D**,**F**) spectra of flavins.

We systematically studied the dependence of the absorption and fluorescence spectra on the pH values for both the S_0_ and S_1_ states of the ten forms of flavins. The pH-dependent absorption and fluorescence spectra of flavins in solution are shown in Figure 5. In Figure 5A, the absorption spectra of flavin quinone in the pH range of 1~8 and 13~14 are consistent with the absorption spectra of ${FL}_{ox}$ and ${FL}_{ox}^{-}$ in Figure 2A, respectively. In addition, the peak positions or intensities of the spectra in the pH range of 9~12 are different from those of ${FL}_{ox}$ and ${FL}_{ox}^{-}$, which is attributed to the coexistence of ${FL}_{ox}$ and ${FL}_{ox}^{-}$ in this pH range (see Figure 4A). In Figure 5B, the fluorescence spectra of flavin quinone in the pH range of 2~14 only have a shark peak when the pH equals to 1; the fluorescence spectrum of flavin quinone has a hump, which can be attributed to the fact that ${FLH}_{ox}^{+}\_N5$ and ${FL}_{ox}$ coexist when the pH equals to 1 (see Figure 4B); and the fluorescence intensity of ${FL}_{ox}$ is stronger than that of ${FLH}_{ox}^{+}\_N5$ (see Figure 3B). In the range of 8~13, the intensity of spectra gradually increases, which can be attributed to the fact that ${FL}_{ox}^{-}$ gradually becomes the dominant form (see Figure 4B). In Figure 5C, the absorption spectra of flavin semiquinone in the pH range of 2~6 and 11~14 are consistent with the absorption spectra of ${HFL}_{sq}^{∙}$ and ${FL}_{sq}^{∙-}$ in Figure 3C, respectively. In the pH range of 1~6, the spectra present a short and wide triple-peak feature. In the pH range of 7~10, the triple-peak feature of the spectra gradually disappears with the increase of pH, because the relative concentration of ${HFL}_{sq}^{∙}$ decreases, and the relative concentration of ${FL}_{sq}^{∙-}$ gradually increases in this pH range (see Figure 4C).The spectrum at pH equal to 1 is different from others, which can be attributed to the coexistence of ${H_{2}FL}_{sq}^{∙+}$ and ${HFL}_{sq}^{∙}$ at pH equal to 1. In Figure 5D, the fluorescence intensity of flavin semiquinone gradually decreases as the pH increases to 7, which is attributed to ${FL}_{sq}^{∙-}$ gradually becoming the dominant existing form (see Figure 4D). In Figure 5E, the spectra in the pH range of 1~4 and 9~14 are consistent with the absorption spectra of $H_{2}{FL}_{red}$ and ${HFL}_{red}^{-}$ in Figure 2E, respectively. In the pH range of 5~8, the shape of the spectrum gradually changes from that of $H_{2}{FL}_{red}$ to that of ${HFL}_{red}^{-}$, because the relative concentration of $H_{2}{FL}_{red}$ decreases in this pH range, while the relative concentration of ${HFL}_{red}^{-}$ increases in this pH range (see Figure 4E). The fluorescence spectra of flavin hydroquinone are short and wide in Figure 5F, which indicates that there is no $H_{2}{FLH}_{red}^{+}$ in the pH range of 1~14 (see Figure 3F and Figure 4F). The $\lambda_{F}$ values and the fluorescence intensity of the fluorescence spectra at the pH range of 1~9 are larger and stronger than those at the pH range of 11~14, and the fluorescence spectrum have two peaks when pH is 10.

## 4. Conclusions

The versatility of flavins is reflected in their various existence forms. Since the available experimental spectra of flavin are limited, we systematically studied the absorption and fluorescence spectra of ten forms of flavin in solution and provided the precise assignment of the excited states in this paper. Every form of flavin has unique spectral characteristics. Most absorption peaks of the absorption spectra of flavin contain the contribution of multiple electronic transitions. In addition, the predicted $\lambda_{max}$ values almost reproduce the existing experimental values. The pKa and $pKa^{*}$ of flavin were calculated, and we found that the pKa values of ${H_{2}FL}_{sq}^{∙+}$ and $H_{2}{FLH}_{red}^{+}$ were greater than their $pKa^{*}$ values, indicating that they are photoacids. The chemical equilibrium between different protonated forms of flavins was obtained, and the existing forms of flavins at different pH were confirmed. The cationic flavin quinone and hydroquinone in the S_0_ state and the cationic flavin semiquinone and hydroquinone in the S_1_ state do not exist throughout the pH range. The cationic flavin quinone in the S_0_ state and the cationic flavin semiquinone in the S_1_ state exist when the pH is lower than 2. For all the redox states of flavins in both the S_0_ and S_1_ states, their dominant forms are their neutral forms before reaching chemical equilibrium, and their anionic forms become the dominant forms after reaching chemical equilibrium when the pH is in the range of 2~14. Moreover, the pH-dependent absorption and fluorescence spectra of flavin were simulated, which provided a spectral basis for determining the presence form of each flavin. For flavin quinone, the pH-dependent absorption spectra retained the double-peaked characteristics in the pH range of 1~14, and the fluorescence intensity of the pH-dependent fluorescence spectra increased as the pH increased. For flavin semiquinone, the triple-peaked absorption spectrum significantly changed to a double-peaked spectrum in the pH range of 7~9, and the fluorescence intensity of the pH-dependent fluorescence spectra decreased as the pH increased. For flavin hydroquinone, the $\lambda_{max}$ values of the absorption spectra underwent a blue shift in the pH range of 4~9. In addition, the fluorescence spectra were short and wide in the pH range of 1~14, and the spectrum had two peaks when the pH was 10.

## Figures and Tables

**Table 1:** Comparison of absorption spectra of flavin between this paper and previous theoretical studies. Experimental results for ${FL}_{ox}$ were taken from Refs. [15,39]; for ${HFL}_{sq}^{∙}$ and ${FL}_{sq}^{∙-}$ from Ref. [40]; and for $H_{2}{FL}_{red}$ and ${HFL}_{red}^{-}$ from Ref. [39].

		TD State Order	Transition	$\lambda_{max}$	*f*	$\lambda_{exp}$
${FL}_{ox}$	This paper	1	$\pi_{2}\to \pi_{3}$	427.5	0.22	445.0
	Ref. [38]		$\pi_{2}\to \pi_{3}$	391.0	0.25	
	Ref. [17]	1		422.7	0.24	
	This paper	2	$\pi_{1}\to \pi_{3}$	358.7	0.31	370.0
	Ref. [38]		$\pi_{1}\to \pi_{3}$	326.0	0.26	
	Ref. [17]	4		345.3	0.25	
${HFL}_{sq}^{∙}$	This paper	1	$\pi_{2}^{\beta }\to \pi_{3}^{\beta }$	568.6	0.11	571.0
	Ref. [38]		$\pi_{2}^{\beta }\to \pi_{3}^{\beta }$	535.0	0.13	
	Ref. [17]	1		581.0	0.13	
	This paper	2	$\pi_{1}^{\beta }\to \pi_{3}^{\beta }$	460.3	0.06	485.0
	Ref. [38]		$\pi_{1}^{\beta }\to \pi_{3}^{\beta }$	406.0	0.06	
	Ref. [17]	3		431.3	0.06	
	This paper	6	$\pi_{3}^{\alpha }\to \pi_{6}^{\alpha }$	340.2	0.08	340.0
	Ref. [38]		$\pi_{0}^{\beta }\to \pi_{3}^{\beta }$	296.0	0.11	
	Ref. [17]	5		360.3	0.09	
${FL}_{sq}^{∙-}$	This paper	2	$\pi_{2}^{\beta }\to \pi_{3}^{\beta }$	450.8	0.04	480.0
	Ref. [38]		$\pi_{2}^{\beta }\to \pi_{3}^{\beta }$	423.0	0.13	
	Ref. [17]	3		437.5	0.14	
	This paper	7	$\pi_{3}^{\alpha }\to \pi_{6}^{\alpha }$	363.1	0.37	370.0
	Ref. [38]		$\pi_{1}^{\beta }\to \pi_{3}^{\beta }$	359.0	0.101	
	Ref. [17]	6		357.6	0.297	
$H_{2}{FL}_{red}$	This paper	1	$\pi_{3}\to \pi_{4}$	392.4	0.06	395.0
	Ref. [38]		$\pi_{3}\to \pi_{4}$	400.0	0.03	
	Ref. [17]	1		411.4	0.03	
${HFL}_{red}^{-}$	This paper	2	$\pi_{3}\to \pi_{5}$	330.4	0.08	342.0
	Ref. [38]		$\pi_{3}\to \pi_{5}$	347.0	0.12	
	Ref. [17]	2		345.4	0.13	

**Table 2:** The pKa and $pKa^{*}$ of the flavins.

Form	pKa		$pKa^{*}$	
	calc.	exp.	calc.	exp.
${FLH}_{ox}^{+}\_N1$	−2.8	0.0 [9]	−4.9	-
${FLH}_{ox}^{+}\_N5$	−10.1	-	0.2	1.7 [9]
${FL}_{ox}$	10.8	10.8 [15]	10.5	10.8 [15]
${H_{2}FL}_{sq}^{∙+}$	0.1	2.3 [45]	−3.1	-
${HFL}_{sq}^{∙}$	8.6	8.5 [28,29]	9.2	-
$H_{2}{FLH}_{red}^{+}$	−3.6	-	−21.1	-
$H_{2}{FL}_{red}$	6.6	6.5 [28,29]	9.7	-

## Data Availability

Data are available from the authors.

## Supplementary Materials

The following are available online at https://www.mdpi.com/article/10.3390/molecules28083315/s1: Table S1: The pKa values under different functional, basis set, and solvation models; Table S2: The TD state order, transition, absorption maximum ($\lambda_{max}$), and oscillator strength (*f*) of absorption spectra of flavins; Table S3: FWHM of absorption and fluorescence spectra of ten forms of flavins; Table S4: The relative concentrations of each form of flavins in S_0_ state at different pH values; Table S5: The relative concentrations of each form of flavins in S_1_ state at different pH values; Table S6: The Cartesian coordinates was optimized equilibrium structures reported in this paper.

## Sample Availability

Samples of the compounds are available from the authors.
