# Supplementary material for: Systematic Theoretical Study on the pH-Dependent Absorption and Fluorescence Spectra of Flavins
Source: Molecules. 2023 Apr 8;28(8):3315. doi: 10.3390/molecules28083315 (PMC10146991; doi:10.3390/molecules28083315)
Supplement: Supplementary file 1 [file molecules-28-03315-s001.zip › molecules-2229184-supplementary.pdf]

Supporting Information

The pH-dependent Absorption and Fluorescence Spectra of Flavins

Jinyu Wang[1] and Ya-Jun Liu[1,2,*]

1. Key Laboratory of Theoretical and Computational Photochemistry, Ministry of Education, College of Chemistry, Beijing Normal University, Beijing 100875, China.
2. Center for Advanced Materials Research, Beijing Normal University, Zhuhai 519087, China.

* Correspondence: yajun.liu@bnu.edu.cn

Contents

1. Additional Table... 2

Table S1 The pKa values under different functional, basis set and solvation models. **2**

Table S2 The TD state order, transition, absorption maximum (λ_{max}) and the oscillator strength (f) of absorption spectra of flavins. States with λ_{max} greater than 300 nm and f greater than 0.01 are listed……………..............**2**

Table S3 FWHM of absorption and fluorescence spectra of ten forms of flavins. **3**

Table S4 The relative concentrations of each form of flavins in S_0 state at different pH values. **5**

Table S5 The relative concentrations of each form of flavins in S_1 state at different pH values. **7**

2. Cartesian coordinates (in Å) 9

1. Additional Table

Table S1 The p*K*a values under different functional, basis set and solvation models.

functional	solvent model	basis set		
		6-31+G**	6-311+G**	6-311++G**
CAM-B3LYP	CPCM	8.5	8.7	8.7
	SMD	8.2	8.3	8.3
M06-2X	CPCM	7.1	7.1	7.1
	SMD	6.6	6.5	6.5
ωB97X-D	CPCM	10.7	10.8	10.8
	SMD	10.2	10.2	10.2

Table S2 The TD state order, transition, absorption maximum (λ_{max}) and the oscillator strength (*f*) of absorption spectra of flavins. States with λ_{max} greater than 300 nm and *f* greater than 0.01 are listed.

State	TD state order	Transition	(λ_{max}/nm)	f
FLH_{ox}^{+}_N1	1	$\pi_2 \rightarrow \pi_3$	410.0	0.03
	2	$\pi_1 \rightarrow \pi_3$	366.0	0.52
FL_{ox}	1	$\pi_2 \rightarrow \pi_3$	427.5	0.22
	2	$\pi_1 \rightarrow \pi_3$	358.7	0.31
FL_{ox}^{-}	2	$\pi_2 \rightarrow \pi_3$	424.0	0.25
	4	$\pi_1 \rightarrow \pi_3$	354.2	0.21
	5	$\pi_0 \rightarrow \pi_3$	338.1	0.15
$H_2FL_{sq}^{\cdot+}$	1	$\pi_2^{\beta} \rightarrow \pi_3^{\beta}$	523.3	0.02
	2	$\pi_3^{\alpha} \rightarrow \pi_4^{\alpha}$	492.0	0.02
	3	$\pi_1^{\beta} \rightarrow \pi_3^{\beta}$	465.2	0.22
	5	$\pi_3^{\alpha} \rightarrow \pi_5^{\alpha}$	342.5	0.16
	6	$\pi_3^{\alpha} \rightarrow \pi_6^{\alpha}$	335.7	0.05
	7	$\pi_2^{\beta} \rightarrow \pi_4^{\beta}$	315.3	0.02
	8	$\pi_0^{\beta} \rightarrow \pi_3^{\beta}$	307.9	0.02

	1	$\pi_2^\beta \rightarrow \pi_3^\beta$	568.6	0.11
	2	$\pi_1^\beta \rightarrow \pi_3^\beta$	460.3	0.06
	3	$\pi_3^\alpha \rightarrow \pi_4^\alpha$	449.2	0.06
$HFL_{sq}^{\cdot}$	5	$\pi_3^\alpha \rightarrow \pi_5^\alpha$	355.5	0.08
	6	$\pi_3^\alpha \rightarrow \pi_6^\alpha$	340.2	0.08
	7	$\pi_0^\beta \rightarrow \pi_3^\beta$	332.1	0.06
	9	$\pi_{-1}^\beta \rightarrow \pi_3^\beta$	321.8	0.03
	10	$\pi_2^\alpha \rightarrow \pi_6^\alpha$	306.0	0.06
	2	$\pi_2^\beta \rightarrow \pi_3^\beta$	450.8	0.04
$FL_{sq}^{\cdot-}$	3	$\pi_3^\alpha \rightarrow \pi_5^\alpha$	431.4	0.03
	4	$\pi_1^\beta \rightarrow \pi_3^\beta$	385.6	0.12
	7	$\pi_3^\alpha \rightarrow \pi_6^\alpha$	363.1	0.37
$H_2FLH_{red}^{+}$	1	$\pi_3 \rightarrow \pi_4$	295.6	0.31
	1	$\pi_3 \rightarrow \pi_4$	392.4	0.06
H_2FL_{red}	2	$\pi_3 \rightarrow \pi_5$	312.0	0.11
	3	$\pi_3 \rightarrow \pi_6$	300.9	0.07
	1	$\pi_3 \rightarrow \pi_4$	370.8	0.03
HFL_{red}^{-}	2	$\pi_3 \rightarrow \pi_5$	330.4	0.08
	3	$\pi_3 \rightarrow \pi_6$	320.3	0.02

Table S3 FWHM of absorption and fluorescence spectra of ten forms of flavins.

Forms	FWHM	
	absorption spectra	fluorescence spectra
FLH_{ox}^{+}_N1	0.5 eV	-
FLH_{ox}^{+}_N5	-	0.5 eV
FL_{ox}	0.4 eV	0.5 eV
FL_{ox}^{-}	0.4 eV	0.5 eV
$H_2FL_{sq}^{\cdot+}$	0.5 eV	0.5 eV
$HFL_{sq}^{\cdot}$	0.5 eV	0.5 eV

$FL_{sq}^{\cdot -}$	0.35 eV	0.5 eV
$H_2FLH_{red}^{+}$	0.5 eV	0.5 eV
H_2FL_{red}	0.7 eV	0.5 eV
HFL_{red}^{-}	0.5 eV	0.5 eV

Table S4 The relative concentrations of each form of flavins in S_0 state at different pH values.

Redox state	form	pH						
		1	2	3	4	5	6	7
quinone	FLH^{+}_{ox}_N1	1.58×10^{-4}	1.58×10^{-5}	1.58×10^{-6}	1.58×10^{-7}	1.58×10^{-8}	1.58×10^{-9}	1.58×10^{-10}
	FL_{ox}	1.00×10^{0}	1.00×10^{0}	1.00×10^{0}	1.00×10^{0}	1.00×10^{0}	1.00×10^{0}	1.00×10^{0}
	FL^{-}_{ox}	1.58×10^{-10}	1.58×10^{-9}	1.58×10^{-8}	1.58×10^{-7}	1.58×10^{-6}	1.58×10^{-4}	1.58×10^{-3}
semiquinone	$H_2FL^{+}_{sq}$	1.12×10^{-1}	1.24×10^{-2}	1.26×10^{-3}	1.26×10^{-4}	1.26×10^{-5}	1.26×10^{-6}	1.23×10^{-7}
	HFL_{sq}	8.88×10^{-1}	9.88×10^{-1}	9.99×10^{-1}	1.00×10^{0}	1.00×10^{0}	9.97×10^{-1}	9.75×10^{-1}
	FL^{-}_{sq}	2.23×10^{-8}	2.48×10^{-7}	2.51×10^{-6}	2.51×10^{-5}	2.51×10^{-4}	2.51×10^{-3}	2.45×10^{-2}
hydroquinone	$H_2FLH^{+}_{red}$	2.51×10^{-5}	2.51×10^{-6}	2.51×10^{-7}	2.51×10^{-8}	2.45×10^{-9}	2.01×10^{-10}	7.15×10^{-12}
	H_2FL_{red}	1.00×10^{0}	1.00×10^{0}	1.00×10^{0}	9.97×10^{-1}	9.75×10^{-1}	7.99×10^{-1}	2.85×10^{-1}
	HFL^{-}_{red}	2.51×10^{-6}	2.51×10^{-5}	2.51×10^{-4}	2.51×10^{-3}	2.45×10^{-2}	2.01×10^{-1}	7.15×10^{-1}
redox state	form	pH						

		8	9	10	11	12	13	14
	$FLH^{+}_{ox\_}N1$	1.58×10^{-11}	1.56×10^{-12}	1.37×10^{-13}	6.13×10^{-15}	9.41×10^{-17}	9.94×10^{-19}	9.99×10^{-21}
quinone	FL_{ox}	9.98×10^{-1}	9.84×10^{-1}	8.63×10^{-1}	3.87×10^{-1}	5.94×10^{-2}	6.27×10^{-3}	6.31×10^{-4}
	FL^{-}_{ox}	1.58×10^{-3}	1.56×10^{-2}	1.37×10^{-1}	6.13×10^{-1}	9.41×10^{-1}	9.94×10^{-1}	9.99×10^{0}
	$H_2FL^{+}_{sq}$	1.01×10^{-8}	3.58×10^{-10}	4.82×10^{-12}	4.99×10^{-14}	5.01×10^{-16}	5.01×10^{-18}	5.01×10^{-20}
semiquinone	HFL_{sq}	7.99×10^{-1}	2.85×10^{-1}	3.83×10^{-2}	3.97×10^{-3}	3.98×10^{-4}	3.98×10^{-5}	3.98×10^{-6}
	FL^{-}_{sq}	2.01×10^{-1}	7.15×10^{-1}	9.62×10^{-1}	9.96×10^{-1}	1.00×10^{0}	1.00×10^{0}	1.00×10^{0}
	$H_2FLH^{+}_{red}$	9.62×10^{-14}	9.96×10^{-16}	1.00×10^{-17}	1.00×10^{-19}	1.00×10^{-21}	1.00×10^{-23}	1.00×10^{-25}
hydroquinone	H_2FL_{red}	3.83×10^{-2}	3.97×10^{-3}	3.98×10^{-4}	3.98×10^{-5}	3.98×10^{-6}	3.98×10^{-7}	3.98×10^{-8}
	HFL^{-}_{red}	9.62×10^{-1}	9.96×10^{-1}	1.00×10^{0}	1.00×10^{0}	1.00×10^{0}	1.00×10^{0}	1.00×10^{0}

Table S5 The relative concentrations of each form of flavins in S_1 state at different pH values.

redox state	form	pH						
		1	2	3	4	5	6	7
quinone	$FLH^{+}_{ox\_}N5$	1.37×10^{-1}	1.56×10^{-2}	1.58×10^{-3}	1.58×10^{-4}	1.58×10^{-5}	1.58×10^{-6}	1.58×10^{-7}
	FL_{ox}	8.63×10^{-1}	9.84×10^{0}	1.00×10^{0}	1.00×10^{0}	1.00×10^{0}	1.00×10^{0}	1.00×10^{0}
	FL^{-}_{ox}	2.73×10^{-10}	3.11×10^{-9}	3.16×10^{-8}	3.16×10^{-7}	3.16×10^{-6}	3.16×10^{-5}	3.16×10^{-4}
semiquinone	$H_2FL^{+}_{sq}$	7.94×10^{-5}	7.94×10^{-6}	7.94×10^{-7}	7.94×10^{-8}	7.94×10^{-9}	7.94×10^{-10}	7.89×10^{-11}
	HFL_{sq}	1.00×10^{0}	1.00×10^{0}	1.00×10^{0}	1.00×10^{0}	1.00×10^{0}	9.99×10^{-1}	9.94×10^{-1}
	FL^{-}_{sq}	6.31×10^{-9}	6.31×10^{-8}	6.31×10^{-7}	6.31×10^{-6}	6.31×10^{-5}	6.31×10^{-4}	6.27×10^{-3}
hydroquinone	$H_2FLH^{+}_{red}$	7.94×10^{-23}	7.94×10^{-24}	7.94×10^{-25}	7.94×10^{-26}	7.94×10^{-27}	7.94×10^{-28}	7.93×10^{-29}
	H_2FL_{red}	1.00×10^{0}	1.00×10^{0}	1.00×10^{0}	1.00×10^{0}	1.00×10^{0}	1.00×10^{0}	9.98×10^{-1}
	HFL^{-}_{red}	2.00×10^{-9}	2.00×10^{-8}	2.00×10^{-7}	2.00×10^{-6}	2.00×10^{-5}	1.99×10^{-4}	1.99×10^{-3}
redox state	form	pH						

		8	9	10	11	12	13	14
	FLH^+_{ox}_N5	1.58×10^{-8}	1.54×10^{-9}	1.20×10^{-10}	3.81×10^{-12}	4.86×10^{-14}	5.00×10^{-16}	5.01×10^{-17}
quinone	FL_{ox}	9.97×10^{-1}	9.69×10^{-1}	7.60×10^{-1}	2.40×10^{-1}	3.07×10^{-2}	3.15×10^{-3}	3.16×10^{-4}
	FL^-_{ox}	3.15×10^{-3}	3.07×10^{-2}	2.40×10^{-1}	7.60×10^{-1}	9.69×10^{-1}	9.97×10^{-1}	1.00×10^{0}
	$H_2FL^+_{sq}$	7.47×10^{-12}	4.87×10^{-13}	1.09×10^{-14}	1.24×10^{-16}	1.26×10^{-18}	1.26×10^{-20}	1.26×10^{-22}
semiquinone	HFL_{sq}	9.41×10^{-1}	6.13×10^{-1}	1.37×10^{-1}	1.56×10^{-2}	1.58×10^{-3}	1.58×10^{-4}	1.58×10^{-5}
	FL^-_{sq}	5.94×10^{-2}	3.87×10^{-1}	8.63×10^{-1}	9.84×10^{-1}	9.98×10^{-1}	1.00×10^{0}	1.00×10^{0}
	$H_2FLH^+_{red}$	7.79×10^{-30}	6.62×10^{-31}	2.65×10^{-32}	3.79×10^{-34}	3.96×10^{-36}	3.98×10^{-38}	3.98×10^{-40}
hydroquinone	H_2FL_{red}	9.80×10^{-1}	8.34×10^{-1}	3.34×10^{-1}	4.77×10^{-2}	4.99×10^{-3}	5.01×10^{-4}	5.01×10^{-5}
	HFL^-_{red}	1.96×10^{-2}	1.66×10^{-1}	6.66×10^{-1}	9.52×10^{-1}	9.95×10^{-1}	9.99×10^{-1}	1.00×10^{0}

2. Cartesian coordinates (in Å)

Table S6. The Cartesian coordinates was optimized equilibrium structures reported in this paper.

gas	FLH_{ox}^{+}_N1-S0			water	FLH_{ox}^{+}_N1-S0		
C	3.410774	0.850475	0.008566	C	3.402238	0.850133	0.011315
C	2.199876	1.501585	-0.028241	C	2.19886	1.508169	-0.041429
C	0.980303	0.787275	-0.040911	C	0.974758	0.7985	-0.062072
C	1.002649	-0.632985	-0.032135	C	0.991648	-0.622911	-0.053659
C	2.232053	-1.299574	0.025238	C	2.218675	-1.296311	0.022555
C	3.419783	-0.579651	0.047509	C	3.404349	-0.581981	0.060921
C	-1.365253	-0.594168	-0.013326	C	-1.361746	-0.590772	-0.038869
C	-1.304296	0.835395	-0.024697	C	-1.304164	0.833455	-0.045929
C	-2.581594	1.625559	-0.017412	C	-2.575481	1.604309	-0.014428
C	-3.820393	-0.531375	0.07624	C	-3.780781	-0.540754	0.094919
H	2.133437	2.585253	-0.043398	H	2.150176	2.592714	-0.060951
H	2.287839	-2.379455	0.081922	H	2.263355	-2.376106	0.079342
C	4.727969	-1.310484	0.11812	C	4.708108	-1.313305	0.160489
H	5.350234	-1.069221	-0.749878	H	5.345105	-1.07651	-0.698607
H	5.291353	-1.009593	1.007271	H	5.253716	-1.002272	1.057801
H	4.585502	-2.391194	0.149654	H	4.553119	-2.392446	0.197435
C	4.702319	1.618459	0.021243	C	4.698735	1.605862	0.029778
H	5.283053	1.397227	0.922147	H	5.274204	1.372984	0.931548
H	5.323879	1.355472	-0.840339	H	5.319413	1.331988	-0.829797
H	4.516074	2.692457	-0.008533	H	4.517196	2.68165	0.000078
C	-0.273367	-2.763462	-0.195929	C	-0.26794	-2.760435	-0.213168
H	0.617891	-3.10147	-0.716295	H	0.627679	-3.100719	-0.72283
H	-0.331023	-3.225698	0.792781	H	-0.337342	-3.194616	0.785751
H	-1.128503	-3.046297	-0.809446	H	-1.11924	-3.04753	-0.827165
N	-0.225335	-1.296151	-0.076071	N	-0.225213	-1.288248	-0.110087
N	-0.177942	1.484896	-0.05259	N	-0.181037	1.495543	-0.074701
N	-2.569356	-1.205765	0.042189	N	-2.565456	-1.211299	0.034516
H	-2.672795	-2.20772	0.152869	H	-2.647249	-2.222292	0.118472
N	-3.735371	0.838866	0.015061	N	-3.715059	0.832082	0.046319
H	-4.623334	1.334367	0.029157	H	-4.609784	1.319143	0.080367
O	-4.836269	-1.163836	0.150265	O	-4.828797	-1.149172	0.182508
O	-2.621544	2.822668	-0.041967	O	-2.62452	2.818386	-0.038298

gas	FLH_{ox}^{+}_N5-S0			water	FLH_{ox}^{+}_N5-S0		
C	-3.385093	-0.846586	-0.000028	C	-3.375519	-0.848959	0.006957
C	-2.173858	-1.499623	-0.000033	C	-2.173592	-1.510194	-0.023749
C	-0.97433	-0.766924	0.000009	C	-0.972254	-0.774842	-0.035

C	-0.962077	0.650191	0.000027	C	-0.960972	0.644081	-0.032219
C	-2.204614	1.302963	0.000055	C	-2.198029	1.307081	0.010029
C	-3.392133	0.585854	0.000032	C	-3.379686	0.5883	0.037671
C	1.454618	0.679696	-0.000015	C	1.438675	0.661557	-0.018539
C	1.379715	-0.763273	0.000022	C	1.37211	-0.771374	-0.026609
C	2.626961	-1.583796	0.000056	C	2.62046	-1.564901	-0.00692
C	3.780412	0.623232	-0.000081	C	3.7456	0.624597	0.056637
H	-2.129843	-2.585851	-0.000085	H	-2.117804	-2.595341	-0.036321
H	-2.247221	2.38485	0.000095	H	-2.239447	2.388398	0.032853
C	-4.701163	1.317913	-0.000019	C	-4.686445	1.315738	0.102723
H	-5.29416	1.049128	-0.880257	H	-5.305978	1.06724	-0.765693
H	-5.294541	1.048611	0.879797	H	-5.247471	1.012572	0.993218
H	-4.55603	2.398403	0.000321	H	-4.533798	2.395252	0.131109
C	-4.675556	-1.615474	-0.000107	C	-4.670422	-1.604668	0.013043
H	-5.276531	-1.372168	0.881489	H	-5.255635	-1.365579	0.906809
H	-5.276739	-1.371685	-0.881426	H	-5.279346	-1.332573	-0.855377
H	-4.492544	-2.690667	-0.000418	H	-4.48916	-2.680417	-0.009548
C	0.240917	2.805275	0.000116	C	0.226061	2.787413	-0.128289
H	-0.26776	3.164982	-0.896708	H	-0.399852	3.093589	-0.96642
H	-0.267419	3.164908	0.897162	H	-0.170272	3.178905	0.810374
H	1.27537	3.137808	-0.000085	H	1.241327	3.135416	-0.277493
N	0.238503	1.333846	0.000052	N	0.240533	1.316381	-0.069
N	0.243851	-1.396323	0.00003	N	0.228978	-1.403478	-0.042075
N	2.575434	1.320997	-0.000076	N	2.573607	1.322519	0.024408
N	3.737133	-0.811314	-0.000083	N	3.727955	-0.785271	0.034629
H	4.644666	-1.271211	-0.000026	H	4.635258	-1.248546	0.056335
O	4.852963	1.157534	-0.000114	O	4.840873	1.174815	0.106022
O	2.549256	-2.794982	0.000161	O	2.607695	-2.785918	-0.023485
H	0.317765	-2.424644	0.000095	H	0.260217	-2.434102	-0.050287

gas		$\mathbf{FL_{ox}^{-}\text{-}S_0}$		water		$\mathbf{FL_{ox}^{-}\text{-}S_0}$	
C	-3.380648	0.85332	-0.000072	C	-3.373168	0.858482	0.000217
C	-2.160777	1.499523	-0.000099	C	-2.162053	1.510048	0.000176
C	-0.947827	0.788126	-0.000045	C	-0.942113	0.797225	-0.00002
C	-0.967083	-0.622159	0.00003	C	-0.959845	-0.617743	-0.000203
C	-2.205634	-1.28348	0.000067	C	-2.196154	-1.285668	-0.000195
C	-3.39487	-0.567067	0.000016	C	-3.382663	-0.570874	0.000022
C	1.435788	-0.630659	0.000074	C	1.42644	-0.62811	-0.000008
C	1.334998	0.83251	0.000011	C	1.337158	0.817521	-0.000074
C	2.625866	1.597732	-0.000065	C	2.614348	1.574596	-0.000211
C	3.755299	-0.633977	0.000285	C	3.732895	-0.622104	0.000409
H	-2.094026	2.583713	-0.000159	H	-2.110478	2.595157	0.000296
H	-2.248151	-2.366197	0.000147	H	-2.237837	-2.367867	-0.000307

H	4.637668	1.229535	-0.000135	H	4.63027	1.239116	-0.000167
C	-4.707847	-1.302315	0.000056	C	-4.692349	-1.303003	0.000019
H	-5.303996	-1.039948	0.880392	H	-5.285892	-1.032958	0.8801
H	-5.303945	-1.040127	-0.880368	H	-5.28564	-1.033305	-0.880346
H	-4.556981	-2.383287	0.00017	H	-4.538053	-2.383286	0.00024
C	-4.669727	1.631956	-0.00012	C	-4.666243	1.624116	0.000491
H	-5.276285	1.398759	-0.881648	H	-5.268424	1.377908	-0.880392
H	-5.276248	1.398921	0.881477	H	-5.267844	1.378189	0.881854
H	-4.472478	2.705423	-0.000223	H	-4.476194	2.699228	0.000271
C	0.241049	-2.767081	0.000103	C	0.2248	-2.769318	-0.000814
H	-0.270544	-3.132125	0.894129	H	-0.293714	-3.117091	0.894096
H	-0.270729	-3.13216	-0.8938	H	-0.294632	-3.116504	-0.895412
H	1.27491	-3.100318	0.000008	H	1.247113	-3.129022	-0.001476
N	0.238626	-1.303754	0.000064	N	0.244028	-1.298195	-0.000385
N	0.228459	1.494244	-0.000063	N	0.226154	1.491029	-0.000077
N	2.547741	-1.306926	0.000164	N	2.562455	-1.306943	0.000366
N	3.730007	0.777525	-0.000021	N	3.722381	0.779653	-0.00023
O	4.827696	-1.196663	-0.000083	O	4.833409	-1.182687	0.000976
O	2.696734	2.805614	-0.000221	O	2.682826	2.797764	-0.000391

gas		$\mathbf{FL}_{ox}^{-}$		water		$\mathbf{FL}_{ox}^{-}$	
C	-3.352845	0.846597	-0.002361	C	3.342633	0.852659	0.019311
C	-2.119471	1.484798	-0.002506	C	2.12622	1.501047	0.01216
C	-0.912083	0.77904	-0.000724	C	0.909304	0.789805	-0.005106
C	-0.941975	-0.631116	0.001453	C	0.928896	-0.620838	-0.017555
C	-2.18615	-1.283069	0.001448	C	2.167198	-1.286501	-0.008985
C	-3.378013	-0.563326	-0.000399	C	3.355497	-0.572146	0.009725
C	1.473031	-0.627356	0.001031	C	-1.462265	-0.626959	-0.004375
C	1.383961	0.845972	0.000728	C	-1.378398	0.825077	-0.01152
C	2.724545	1.58294	0.003152	C	-2.684719	1.556632	-0.024861
C	3.814235	-0.51881	-0.004156	C	-3.768423	-0.525167	0.043784
H	-2.047741	2.569426	-0.003887	H	2.074543	2.586476	0.019608
H	-2.229407	-2.36695	0.002805	H	2.208533	-2.369152	-0.013431
C	-4.695559	-1.296576	-0.000315	C	4.666904	-1.304495	0.020062
H	-5.295413	-1.037909	0.879925	H	5.268302	-1.036775	-0.855536
H	-5.294567	-1.039655	-0.881637	H	5.255028	-1.037694	0.904753
H	-4.542785	-2.378474	0.000848	H	4.512658	-2.385099	0.018167
C	-4.634717	1.641076	-0.004088	C	4.632786	1.625131	0.036713
H	-5.247418	1.418651	-0.885881	H	5.227749	1.381078	0.923294
H	-5.248603	1.420414	0.877326	H	5.246849	1.387359	-0.838482
H	-4.422171	2.712656	-0.005019	H	4.437609	2.699617	0.038475
C	0.27058	-2.767571	0.006301	C	-0.261392	-2.768773	-0.074933
H	-0.237711	-3.149776	0.898375	H	0.30479	-3.100633	-0.94682
H	-0.232683	-3.153518	-0.887062	H	0.200079	-3.153099	0.837147

H	1.313402	-3.079728	0.009524	H	-1.284014	-3.122383	-0.144941
N	0.255938	-1.315258	0.003593	N	-0.275885	-1.302726	-0.038546
N	0.275205	1.499588	-0.000283	N	-0.266578	1.490562	-0.013096
N	2.575945	-1.275534	-0.000705	N	-2.600217	-1.276886	0.026521
N	3.843322	0.841346	-0.000439	N	-3.814262	0.844222	0.00342
O	4.832125	-1.204682	-0.010467	O	-4.849274	-1.160259	0.093004
O	2.687748	2.814466	0.008235	O	-2.675601	2.809168	-0.062611

gas		**$H_2FL_{aq}^{+}$-S₀**		water		**$H_2FL_{aq}^{+}$-S₀**	
C	3.436659	0.820024	0.019758	C	3.430471	0.819329	0.030139
C	2.228928	1.492637	-0.05136	C	2.22738	1.4971	-0.051801
C	1.015621	0.795998	-0.076785	C	1.015	0.798286	-0.092695
C	1.00436	-0.616199	-0.045985	C	1.000492	-0.612202	-0.066162
C	2.224027	-1.289524	0.050061	C	2.21683	-1.291538	0.038697
C	3.431665	-0.598603	0.082746	C	3.422094	-0.599485	0.086741
C	-1.38549	-0.596956	-0.032995	C	-1.379158	-0.586403	-0.054675
C	-1.365581	0.787261	-0.048033	C	-1.362668	0.803255	-0.071242
C	-2.595438	1.579168	-0.008862	C	-2.590783	1.566216	-0.008819
C	-3.83597	-0.558928	0.117593	C	-3.798312	-0.568493	0.131538
H	-0.246814	2.476459	-0.144544	H	-0.201568	2.48733	-0.168403
H	2.208631	2.578703	-0.084163	H	2.199671	2.582968	-0.081088
H	2.248862	-2.370456	0.119303	H	2.237446	-2.372803	0.096707
H	-4.637995	1.319083	0.08979	H	-4.628915	1.294204	0.127755
H	-2.676059	-2.222686	0.193355	H	-2.641138	-2.232272	0.155351
C	4.726555	-1.352869	0.187776	C	4.715604	-1.353205	0.199974
H	5.371971	-1.139169	-0.670342	H	5.370043	-1.128791	-0.649318
H	5.278764	-1.058774	1.086093	H	5.257408	-1.06328	1.106679
H	4.559116	-2.429998	0.228193	H	4.540363	-2.430128	0.22846
C	4.733036	1.579229	0.04326	C	4.729126	1.572023	0.067082
H	5.296038	1.364157	0.95692	H	5.294529	1.332402	0.973635
H	5.366885	1.295007	-0.802618	H	5.35935	1.299949	-0.786437
H	4.562138	2.655343	-0.00618	H	4.554939	2.649266	0.040511
C	-0.241557	-2.757977	-0.296744	C	-0.247517	-2.753229	-0.28695
H	0.590172	-3.036767	-0.941567	H	0.604887	-3.052488	-0.89201
H	-0.156206	-3.273263	0.66432	H	-0.201773	-3.230044	0.694689
H	-1.154377	-3.056625	-0.810951	H	-1.143701	-3.059344	-0.822988
N	-0.228822	-1.303051	-0.119269	N	-0.23105	-1.291423	-0.151557
N	-0.188275	1.458825	-0.122658	N	-0.180693	1.467951	-0.150194
N	-2.594735	-1.224424	0.046584	N	-2.582893	-1.223151	0.047445
N	-3.753009	0.820789	0.049172	N	-3.738809	0.802432	0.071618
O	-4.861367	-1.173848	0.226427	O	-4.84679	-1.182317	0.248929
O	-2.578398	2.790654	-0.038552	O	-2.620557	2.792622	-0.03339

gas		**HFL$_{sq}$-S$_0$**		water		**HFL$_{sq}$-S$_0$**	
C	-3.41208	0.821144	0.000037	C	3.405709	0.826195	0.009272
C	-2.19518	1.489691	0.000034	C	2.195139	1.498168	-0.02184
C	-0.984654	0.794495	0.000012	C	0.983904	0.796306	-0.035135
C	-0.969109	-0.615596	-0.000007	C	0.969626	-0.612655	-0.024934
C	-2.200376	-1.280018	0.000004	C	2.197347	-1.284604	0.017718
C	-3.410593	-0.58871	0.000025	C	3.403311	-0.590798	0.036013
C	1.458353	-0.63604	0.000011	C	-1.44408	-0.624098	-0.017508
C	1.401702	0.783071	0.00002	C	-1.396865	0.787234	-0.02376
C	2.626071	1.555097	-0.000003	C	-2.621513	1.53714	0.000215
C	3.779099	-0.648065	0.000227	C	-3.754298	-0.641322	0.048446
H	0.291896	2.471194	-0.000008	H	-0.244731	2.478244	-0.062946
H	-2.170638	2.576795	0.000045	H	2.163281	2.584608	-0.035998
H	-2.223792	-2.363226	0.000005	H	2.221968	-2.367341	0.039028
H	4.650917	1.23067	-0.00007	H	-4.64783	1.222578	0.053872
C	-4.710337	-1.349455	0.000025	C	4.702243	-1.345603	0.083557
H	-5.313249	-1.103826	0.880865	H	5.325043	-1.108278	-0.785853
H	-5.313106	-1.104055	-0.88098	H	5.280316	-1.075743	0.974072
H	-4.535722	-2.4271	0.000178	H	4.527504	-2.423248	0.099106
C	-4.706956	1.589838	0.000069	C	4.702389	1.586362	0.016247
H	-5.311202	1.349071	-0.881021	H	5.293859	1.347102	0.906386
H	-5.311006	1.349313	0.881362	H	5.313346	1.325784	-0.85477
H	-4.524031	2.666192	-0.000098	H	4.521529	2.663083	0.000924
C	0.24041	-2.76609	-0.000101	C	-0.22434	-2.76283	-0.110102
H	-0.270896	-3.136189	0.893631	H	0.383111	-3.083943	-0.958319
H	-0.271055	-3.136103	-0.893776	H	0.196825	-3.158378	0.817616
H	1.273523	-3.103545	-0.000202	H	-1.237944	-3.127018	-0.232498
N	0.247675	-1.308438	-0.000044	N	-0.25133	-1.300595	-0.063446
N	0.226746	1.456292	0.00001	N	-0.218079	1.45988	-0.056235
N	2.575527	-1.319519	0.00005	N	-2.582667	-1.315746	0.02174
N	3.749292	0.768642	0.00003	N	-3.74596	0.754137	0.032856
O	4.859957	-1.201466	-0.000168	O	-4.858953	-1.211618	0.087597
O	2.631992	2.783441	-0.000129	O	-2.66873	2.776849	-0.009366

gas		**FL$^{-}_{sq}$-S$_0$**		water		**FL$^{-}_{sq}$-S$_0$**	
C	3.40669	0.830756	0.000064	C	3.402626	0.836567	0.003347
C	2.189379	1.499073	0.000048	C	2.187119	1.503379	-0.01106
C	0.94585	0.827992	0.000018	C	0.947777	0.827567	-0.015289
C	0.974275	-0.598393	-0.000021	C	0.968887	-0.591643	-0.009799
C	2.199221	-1.268015	0.000019	C	2.193326	-1.266616	0.011628
C	3.417529	-0.576162	0.000057	C	3.406783	-0.576917	0.018723
C	-1.447092	-0.575573	-0.000017	C	-1.435672	-0.585289	-0.007419
C	-1.356559	0.843666	0.000057	C	-1.36092	0.831428	-0.009862
C	-2.620565	1.572317	0.000145	C	-2.611885	1.545559	-0.000566

C	-3.75867	-0.660079	0.000003	C	-3.744299	-0.637832	0.023144
H	2.149839	2.585302	0.00007	H	2.161233	2.590459	-0.019502
H	2.222897	-2.352877	0.000017	H	2.217958	-2.350377	0.023263
H	-4.637227	1.199341	-0.000017	H	-4.638337	1.217139	0.021858
C	4.718711	-1.337501	0.000146	C	4.705965	-1.335783	0.041126
H	5.329555	-1.100639	-0.880411	H	5.320473	-1.097435	-0.834518
H	5.329142	-1.101124	0.881126	H	5.300966	-1.080173	0.925293
H	4.5387	-2.416116	-0.000192	H	4.528258	-2.413677	0.049792
C	4.702881	1.603556	0.000092	C	4.699318	1.601797	0.003397
H	5.312699	1.369099	0.881295	H	5.300702	1.364622	0.88798
H	5.312502	1.369481	-0.881351	H	5.307826	1.350987	-0.872599
H	4.510795	2.679374	0.000342	H	4.514557	2.678484	-0.005848
C	-0.245783	-2.726567	-0.000282	C	-0.226444	-2.741084	-0.057312
H	0.270843	-3.104916	-0.891175	H	0.341997	-3.082928	-0.926017
H	0.270513	-3.105157	0.890704	H	0.240889	-3.1251	0.854001
H	-1.280524	-3.059573	-0.000501	H	-1.243644	-3.110514	-0.123819
N	-0.243997	-1.278151	-0.000117	N	-0.251592	-1.282023	-0.031361
N	-0.200515	1.547685	0.000033	N	-0.207346	1.551158	-0.023543
N	-2.557889	-1.303181	-0.00005	N	-2.573137	-1.297335	0.011815
N	-3.736477	0.739623	0.000056	N	-3.737724	0.748122	0.013615
O	-4.850124	-1.231263	-0.000178	O	-4.855606	-1.218932	0.041953
O	-2.763976	2.791957	0.000011	O	-2.739365	2.787964	-0.00619

gas		$H_2FLH^+_{red}$-S_0		water		$H_2FLH^+_{red}$-S_0	
C	3.399887	-0.799276	-0.14451	C	3.393022	-0.797322	-0.15123
C	2.209665	-1.471065	0.12201	C	2.208834	-1.476935	0.127397
C	1.03467	-0.764198	0.324215	C	1.037868	-0.771575	0.346334
C	0.984259	0.62769	0.26751	C	0.986419	0.617722	0.291182
C	2.178207	1.297695	-0.008868	C	2.170462	1.298226	0.000002
C	3.375502	0.609502	-0.206748	C	3.36547	0.609885	-0.212369
C	-1.379525	0.606403	0.215799	C	-1.368431	0.586927	0.21928
C	-1.390231	-0.759871	0.256713	C	-1.377597	-0.788336	0.234409
C	-2.544786	-1.55949	-0.042738	C	-2.542149	-1.548311	-0.05373
C	-3.760689	0.565845	-0.379296	C	-3.717292	0.583693	-0.390292
H	2.202366	-2.557507	0.179107	H	2.19323	-2.56257	0.183826
H	2.181954	2.378366	-0.09627	H	2.167107	2.379122	-0.084865
C	4.637576	1.372162	-0.502727	C	4.622441	1.373475	-0.525405
H	5.395692	1.181231	0.263502	H	5.396624	1.172131	0.222675
H	5.063624	1.06255	-1.462096	H	5.028766	1.0708	-1.496415
H	4.455502	2.446988	-0.540796	H	4.433077	2.448306	-0.547244
C	4.677843	-1.560768	-0.363294	C	4.672167	-1.551712	-0.383736
H	5.082244	-1.368972	-1.362232	H	5.071311	-1.346514	-1.382939
H	5.442831	-1.259707	0.359262	H	5.440246	-1.251527	0.337148
H	4.521238	-2.635933	-0.262873	H	4.512125	-2.627712	-0.290415

C	-0.258878	2.774398	0.571381	C	-0.263402	2.756434	0.590511
H	0.570393	3.071823	1.212869	H	0.579992	3.059297	1.209187
H	-0.17185	3.27894	-0.396623	H	-0.200854	3.242375	-0.3872
H	-1.176654	3.078171	1.076322	H	-1.168994	3.065352	1.112066
N	-0.242356	1.318654	0.433636	N	-0.24476	1.297011	0.469133
N	-0.204205	-1.495464	0.663151	N	-0.195591	-1.498194	0.692543
N	-2.556657	1.235374	-0.07113	N	-2.53473	1.233072	-0.084667
N	-3.679088	-0.815533	-0.334297	N	-3.66194	-0.784826	-0.361939
O	-4.7538	1.179164	-0.663236	O	-4.731339	1.206656	-0.67323
O	-2.515018	-2.779371	-0.027506	O	-2.595315	-2.78173	-0.029297
H	-2.600168	2.232579	-0.241108	H	-2.558507	2.241419	-0.211176
H	-4.532158	-1.319247	-0.558586	H	-4.524365	-1.279832	-0.577278
H	-0.258734	-2.412782	0.188249	H	-0.186569	-2.43559	0.260819
H	-0.23061	-1.71021	1.671004	H	-0.237678	-1.656928	1.712414

gas		$\mathbf{H_2FL_{red}}$**-S0**		water		$\mathbf{H_2FL_{red}}$**-S0**	
C	3.383093	0.795065	0.142841	C	3.371764	0.798317	0.14924
C	2.198887	1.475868	-0.159768	C	2.190922	1.480831	-0.164967
C	1.005814	0.801561	-0.385028	C	1.005222	0.799696	-0.410681
C	0.987988	-0.60062	-0.292737	C	0.983776	-0.59891	-0.328125
C	2.152519	-1.278988	0.043495	C	2.145012	-1.282743	0.016636
C	3.360364	-0.600042	0.251172	C	3.347286	-0.60078	0.244586
C	-1.384057	-0.555152	-0.261608	C	-1.375634	-0.557226	-0.268667
C	-1.362943	0.800441	-0.324909	C	-1.35625	0.811564	-0.315813
C	-2.524919	1.569857	0.023509	C	-2.511594	1.555311	0.034748
C	-3.72575	-0.575794	0.424591	C	-3.691982	-0.571358	0.428143
H	-0.220127	2.452058	-0.55231	H	-0.186649	2.449011	-0.536035
H	2.207118	2.56065	-0.236941	H	2.191988	2.566611	-0.231227
H	2.139955	-2.359666	0.146179	H	2.132442	-2.363587	0.1111
H	-4.495817	1.301096	0.585811	H	-4.478556	1.29567	0.619937
H	-2.549149	-2.216522	0.257639	H	-2.539629	-2.225151	0.209537
C	4.613021	-1.367493	0.588719	C	4.595827	-1.367358	0.593914
H	5.388144	-1.223874	-0.172467	H	5.388418	-1.194814	-0.142896
H	5.037105	-1.039587	1.544156	H	4.99318	-1.057239	1.566893
H	4.410335	-2.438545	0.661768	H	4.396126	-2.440739	0.633029
C	4.663227	1.560486	0.356648	C	4.64882	1.558445	0.38893
H	5.062073	1.39636	1.363796	H	5.040151	1.367591	1.394457
H	5.437761	1.244578	-0.350949	H	5.428057	1.254472	-0.319049
H	4.506244	2.633455	0.227337	H	4.490134	2.633889	0.281725
C	-0.266069	-2.717771	-0.663723	C	-0.274226	-2.728681	-0.621873
H	0.572964	-3.034618	-1.285844	H	0.57531	-3.07708	-1.208735
H	-0.188987	-3.222778	0.31079	H	-0.221107	-3.163507	0.383219
H	-1.180478	-3.033325	-1.170591	H	-1.175593	-3.072224	-1.130317
N	-0.243402	-1.274438	-0.559846	N	-0.2522	-1.270848	-0.595953

N	-0.185468	1.456374	-0.744319	N	-0.18746	1.463371	-0.78741
N	-2.540782	-1.219872	0.09557	N	-2.523155	-1.215452	0.103632
N	-3.652087	0.79604	0.342355	N	-3.629825	0.792262	0.375355
O	-4.7226	-1.18777	0.755214	O	-4.709104	-1.188802	0.746906
O	-2.575152	2.791455	0.029297	O	-2.585828	2.79619	0.030914

gas		$\mathbf{HFL_{red}^{-}}$-S₀		water		$\mathbf{HFL_{red}^{-}}$-S₀	
C	3.382735	0.796941	0.113839	C	3.346223	0.802718	0.141508
C	2.181058	1.4777	-0.123901	C	2.159832	1.478562	-0.167503
C	0.975705	0.809851	-0.317349	C	0.974503	0.79363	-0.408712
C	0.954556	-0.606445	-0.250473	C	0.951104	-0.608175	-0.326531
C	2.148081	-1.274392	0.013829	C	2.124125	-1.281671	0.010541
C	3.366543	-0.595841	0.185261	C	3.325944	-0.595752	0.23404
C	-1.461589	-0.587926	-0.211738	C	-1.436528	-0.592652	-0.258712
C	-1.404378	0.790861	-0.261504	C	-1.387781	0.79388	-0.309343
C	-2.562323	1.543088	0.04058	C	-2.52893	1.527724	0.048866
C	-3.708059	-0.660232	0.31243	C	-3.646915	-0.634017	0.397566
H	-0.254098	2.452128	-0.408723	H	-0.218111	2.43533	-0.53636
H	2.181259	2.564916	-0.176426	H	2.15578	2.56471	-0.233399
H	2.148388	-2.357006	0.084182	H	2.122247	-2.362752	0.100398
H	-4.536051	1.217192	0.519489	H	-4.478027	1.232987	0.649523
C	4.633287	-1.371788	0.44868	C	4.579089	-1.359551	0.575819
H	5.389671	-1.193145	-0.326121	H	5.369376	-1.182393	-0.162598
H	5.088846	-1.093548	1.407439	H	4.979716	-1.053419	1.548923
H	4.432555	-2.446305	0.47572	H	4.383281	-2.43396	0.611271
C	4.667628	1.565575	0.29666	C	4.621117	1.568773	0.377711
H	5.111043	1.383521	1.283475	H	5.016317	1.382936	1.382851
H	5.420035	1.278151	-0.448304	H	5.401382	1.268821	-0.33107
H	4.495978	2.641061	0.200784	H	4.457346	2.643537	0.269089
C	-0.266147	-2.726828	-0.425866	C	-0.273401	-2.736605	-0.548694
H	0.448698	-3.122037	-1.155634	H	0.538271	-3.11235	-1.173468
H	0.002819	-3.103268	0.573643	H	-0.14492	-3.1164	0.473165
H	-1.272676	-3.067108	-0.658609	H	-1.215782	-3.105986	-0.944679
N	-0.258665	-1.282174	-0.481328	N	-0.273299	-1.282002	-0.599936
N	-0.206672	1.464097	-0.633151	N	-0.216583	1.449636	-0.786253
N	-2.54463	-1.318888	0.060228	N	-2.524529	-1.306691	0.08339
N	-3.666037	0.745197	0.315089	N	-3.62305	0.74948	0.393916
O	-4.789683	-1.209669	0.547946	O	-4.725135	-1.208328	0.705673
O	-2.631194	2.787724	0.06212	O	-2.619233	2.786428	0.057977

water		$\mathbf{FLH_{ox}^{+}}$_N5-S₁	
C	-3.410805	-0.820546	0.005953
C	-2.167375	-1.497881	-0.041588

C	-0.965454	-0.807943	-0.057353
C	-0.960492	0.606628	-0.041507
C	-2.210324	1.277687	0.017908
C	-3.420894	0.594948	0.047145
C	1.433493	0.606505	-0.046736
C	1.408774	-0.812742	-0.040547
C	2.646912	-1.550656	0.0236
C	3.767112	0.633601	0.032942
H	-2.149702	-2.581388	-0.064551
H	-2.226143	2.357436	0.044783
C	-4.71339	1.353586	0.118207
H	-5.346936	1.126542	-0.744924
H	-5.280665	1.074022	1.011528
H	-4.535078	2.428709	0.143598
C	-4.674357	-1.605013	0.012036
H	-5.271611	-1.364869	0.90004
H	-5.297785	-1.332612	-0.848762
H	-4.48575	-2.67749	-0.011616
C	0.226465	2.774443	-0.121138
H	-0.353622	3.104178	-0.983055
H	-0.212171	3.162722	0.798947
H	1.24731	3.121906	-0.211477
N	0.233244	1.299582	-0.080319
N	0.235177	-1.480489	-0.079983
N	2.555642	1.301256	-0.021959
N	3.771028	-0.749963	0.071207
H	4.675081	-1.211855	0.111535
O	4.82589	1.25895	0.050346
O	2.707508	-2.783124	0.039315
H	0.252584	-2.498441	-0.077782

Water		**FL_{ox}-S_1**	
C	3.397558	0.835632	-0.000007
C	2.155597	1.508516	-0.000114
C	0.933866	0.84254	-0.000135
C	0.964555	-0.592069	-0.00012
C	2.209697	-1.267461	0.00003
C	3.418909	-0.586297	0.000105
C	-1.424532	-0.568778	-0.000212
C	-1.366954	0.85787	-0.000209
C	-2.631927	1.563633	-0.000523
C	-3.754054	-0.625353	0.000445
H	2.141448	2.592063	-0.000184

H	2.226705	-2.348082	0.000099
H	-4.662724	1.212857	0.000439
C	4.7173	-1.341417	0.000283
H	5.321225	-1.092506	-0.878604
H	5.321062	-1.092375	0.879245
H	4.541434	-2.417889	0.000347
C	4.663671	1.622686	-0.000015
H	5.275967	1.373377	0.875097
H	5.276009	1.37328	-0.875071
H	4.470717	2.695196	-0.000082
C	-0.227544	-2.754071	-0.00033
H	0.283808	-3.118093	-0.892763
H	0.283444	-3.118181	0.892278
H	-1.252654	-3.10115	-0.00055
N	-0.227036	-1.28197	-0.000255
N	-0.223697	1.568785	-0.000221
N	-2.539419	-1.279509	-0.000082
N	-3.759577	0.749318	0.000164
O	-4.815341	-1.261063	0.000076
O	-2.764088	2.797687	0.00035

water		**FL_{ox}^{-}-S1**	
C	3.362118	0.84001	-0.000006
C	2.131999	1.508202	-0.000012
C	0.902941	0.84011	-0.000014
C	0.936322	-0.601676	-0.000002
C	2.176725	-1.272417	0
C	3.383726	-0.585255	-0.000003
C	-1.451456	-0.564461	0.000008
C	-1.40383	0.851372	-0.000017
C	-2.697351	1.532613	-0.000049
C	-3.791092	-0.540447	0.000027
H	2.112917	2.592016	-0.000015
H	2.197782	-2.353252	0.000005
C	4.684954	-1.337146	-0.000002
H	5.289876	-1.087872	-0.878468
H	5.289869	-1.087882	0.878472
H	4.512705	-2.414514	-0.000008
C	4.637863	1.622917	-0.000003
H	5.248561	1.378127	0.876532
H	5.248558	1.378138	-0.876543
H	4.445389	2.69617	0.000003
C	-0.264361	-2.756069	0.000026

H	0.246472	-3.125117	-0.89137
H	0.246482	-3.125102	0.891422
H	-1.291523	-3.098686	0.000034
N	-0.259019	-1.286972	0.000011
N	-0.252206	1.562922	-0.000025
N	-2.579216	-1.258094	0.000019
N	-3.847453	0.803544	-0.000026
O	-4.839642	-1.246963	0.000068
O	-2.749263	2.797316	-0.000094

water		$\mathbf{H_2FL^{+}_{aq}}$-S1	
C	3.446431	0.783125	0.030431
C	2.196498	1.477167	-0.225334
C	0.989666	0.811946	-0.362678
C	0.982576	-0.585385	-0.255285
C	2.194738	-1.258705	0.041669
C	3.43038	-0.597227	0.166653
C	-1.383437	-0.547658	-0.179119
C	-1.385281	0.818158	-0.259777
C	-2.563904	1.557438	0.048558
C	-3.712389	-0.58806	0.486377
H	-0.206632	2.482094	-0.493392
H	2.218818	2.555004	-0.34004
H	2.166877	-2.330697	0.187258
H	-4.538044	1.268771	0.593377
H	-2.507752	-2.226236	0.291274
C	4.681439	-1.38304	0.448544
H	5.396104	-1.294741	-0.375984
H	5.182282	-1.014334	1.348569
H	4.454497	-2.440325	0.589744
C	4.700134	1.581272	0.141889
H	5.121637	1.488521	1.151141
H	5.466687	1.201093	-0.542424
H	4.528975	2.637136	-0.06515
C	-0.251316	-2.731581	-0.631462
H	0.6057	-3.024661	-1.233922
H	-0.231757	-3.269093	0.320535
H	-1.148079	-2.999411	-1.188404
N	-0.217825	-1.276739	-0.442022
N	-0.201659	1.478333	-0.642083
N	-2.506783	-1.215324	0.201145
N	-3.674579	0.775571	0.387611

O	-4.711453	-1.227054	0.796712
O	-2.65097	2.792292	0.0134

Water		$\mathbf{HFL_{sq}}$-S1	
C	-0.18728	0.223461	0.000062
C	-1.439099	0.912953	-0.000071
C	-2.644515	0.251282	-0.000142
C	-2.641037	-1.174858	-0.000069
C	-1.401186	-1.847864	0.000094
C	-0.175864	-1.179598	0.000167
C	-5.070713	-1.164376	0.000055
C	-5.05644	0.229842	-0.000056
C	-6.285127	0.92526	0.000098
C	-7.397849	-1.261928	0.000487
H	-1.438172	1.997263	-0.000158
H	-1.393105	-2.928642	0.000163
H	-8.321669	0.570862	0.000549
C	1.113963	-1.956689	0.000339
H	1.722835	-1.722398	-0.878994
H	1.722658	-1.722309	0.879771
H	0.920222	-3.030655	0.000375
C	1.080604	1.01461	0.000074
H	1.69295	0.770367	0.876096
H	1.69301	0.770283	-0.875883
H	0.886086	2.087282	0.000014
C	-3.83186	-3.345725	-0.000373
H	-3.321754	-3.716262	-0.892034
H	-3.322059	-3.716488	0.891372
H	-4.855476	-3.69938	-0.000589
N	-3.841801	-1.879587	-0.000215
N	-3.852047	0.932333	-0.000371
N	-6.175903	-1.894812	0.000235
N	-7.417758	0.110322	0.000398
O	-8.457125	-1.913722	0.000596
O	-6.402587	2.174054	0.000191
H	-3.872196	1.941029	-0.000394

water		$\mathbf{FL_{sq}^{-}}$-S1	
C	-3.399393	0.854584	0.000143

C	-2.180238	1.49539	0.000235
C	-0.949112	0.802245	0.000273
C	-0.962508	-0.650408	0.000186
C	-2.224003	-1.30472	0.000077
C	-3.405966	-0.595932	0.000069
C	1.429966	-0.63017	0.000504
C	1.34579	0.844475	0.000296
C	2.600738	1.590978	0.000044
C	3.742485	-0.633798	0.000638
H	-2.134808	2.580197	0.000257
H	-2.268604	-2.385523	0.000005
H	4.624517	1.264341	-0.000454
C	-4.716943	-1.332875	-0.000041
H	-5.319375	-1.073042	0.877349
H	-5.319264	-1.072982	-0.877489
H	-4.562408	-2.413204	-0.000069
C	-4.689935	1.631204	0.000105
H	-5.306027	1.406599	-0.878431
H	-5.306083	1.406593	0.878601
H	-4.49033	2.705045	0.000116
C	0.228176	-2.785885	0.000089
H	-0.280301	-3.156271	0.890908
H	-0.280268	-3.156092	-0.890822
H	1.256544	-3.125777	0.000072
N	0.224309	-1.312947	0.000231
N	0.215723	1.498875	0.000206
N	2.606952	-1.309538	0.000558
N	3.725649	0.798791	-0.000098
O	4.897115	-1.156883	-0.000362
O	2.70136	2.833537	-0.00047

Water		$\mathbf{H_2FLH^+_{red}}$-S_1	
C	3.444628	-0.780799	-0.194324
C	2.227254	-1.4664	-0.138695
C	1.035827	-0.78894	-0.00691
C	0.999049	0.642857	0.077048
C	2.249195	1.322462	0.077605
C	3.441023	0.649589	-0.069713
C	-1.350385	0.499999	-0.263314
C	-1.4257	-0.81213	0.208709
C	-2.635784	-1.481577	0.457524
C	-3.782302	0.618476	-0.217433
H	2.219331	-2.549894	-0.181765

H	2.258846	2.401693	0.133625
C	4.734535	1.410393	-0.123096
H	5.414542	1.078294	0.667361
H	5.247564	1.238522	-1.074386
H	4.565384	2.481239	-0.008142
C	4.723907	-1.53215	-0.367467
H	5.243254	-1.20814	-1.277089
H	5.410819	-1.33478	0.463022
H	4.548403	-2.605952	-0.430918
C	-0.314456	2.716593	0.385971
H	0.475329	3.029251	1.063669
H	-0.260397	3.296333	-0.540489
H	-1.274224	2.896021	0.866621
N	-0.187098	1.284074	0.103467
N	-0.183641	-1.56963	0.257171
N	-2.550242	1.170616	-0.467711
N	-3.769014	-0.649164	0.327363
O	-4.830403	1.215194	-0.4677
O	-2.774497	-2.695387	0.728682
H	-2.56357	2.057917	-0.958286
H	-4.674404	-1.07677	0.48581
H	-0.234675	-2.345847	-0.42299
H	-0.098924	-2.02823	1.179949

water		$\mathbf{H_2FL_{red}}$-S1	
C	-0.807865	-0.296727	0.000005
C	0.415414	0.348913	0.000062
C	1.628931	-0.357472	0.000088
C	1.628531	-1.800163	0.00006
C	0.367723	-2.441396	-0.00002
C	-0.82249	-1.731349	-0.000047
C	4.020964	-1.786452	0.000182
C	4.030522	-0.35962	0.000111
C	5.219884	0.383802	-0.000064
C	6.462356	-1.769385	0.000154
H	0.461374	1.433558	0.000085
H	0.318211	-3.521112	-0.00007
H	7.286609	0.10088	-0.000196
C	-2.132289	-2.47237	-0.00014
H	-2.736009	-2.217467	0.877705
H	-2.735925	-2.217401	-0.878025
H	-1.972518	-3.551951	-0.000173
C	-2.090511	0.490238	-0.000005

H	-2.70526	0.263622	-0.878348
H	-2.705285	0.263597	0.878315
H	-1.887095	1.562879	0.000014
C	2.776283	-3.95377	0.000162
H	2.257053	-4.309785	0.891578
H	2.257435	-4.309851	-0.891453
H	3.773024	-4.377884	0.000406
N	2.820537	-2.486216	0.000136
N	2.823421	0.298318	0.000118
N	5.265487	-2.423608	0.00015
N	6.402861	-0.392644	-0.000065
O	7.544339	-2.376128	-0.00007
O	5.316268	1.641263	-0.000044
H	2.841061	1.313313	0.000069
H	5.344499	-3.430993	0.000285

water		$\mathbf{HFL_{red}^{-}}$-S1	
C	-0.809647	-0.297041	0.000001
C	0.416898	0.353525	0.000008
C	1.631422	-0.343291	-0.000005
C	1.641846	-1.78963	0.000022
C	0.379243	-2.436638	-0.000038
C	-0.815612	-1.734841	-0.000052
C	4.060587	-1.79006	-0.000101
C	4.031749	-0.347694	-0.000017
C	5.234082	0.384113	0.000036
C	6.381888	-1.820071	-0.000236
H	0.455655	1.439377	0.00003
H	0.338211	-3.517766	-0.000076
H	7.277148	0.065777	0.000102
C	-2.123204	-2.483737	-0.00012
H	-2.731449	-2.236457	0.877527
H	-2.731394	-2.236393	-0.877786
H	-1.956734	-3.562928	-0.000155
C	-2.095104	0.487626	0.000032
H	-2.713992	0.266469	-0.878086
H	-2.713976	0.26642	0.878149
H	-1.892523	1.561126	0.000061
C	2.826701	-3.930361	0.000214
H	2.312393	-4.300008	0.889808
H	2.312935	-4.300213	-0.889612
H	3.849891	-4.286669	0.000568
N	2.842492	-2.464346	0.000081

N	2.833201	0.31354	0.000031
N	5.233393	-2.48339	-0.000143
N	6.381933	-0.404083	-0.000027
O	7.52834	-2.376129	0.000174
O	5.329614	1.647966	0.000145
H	2.851968	1.327909	0.000074
